# Deducing Underlying Mechanisms from Protein Recruitment Data

**DOI:** 10.1371/journal.pone.0066590

**Published:** 2013-06-27

**Authors:** Laurin Lengert, Barbara Drossel

**Affiliations:** Institute for Condensed Matter Physics, TU Darmstadt, Darmstadt, Germany; Universidad de Granada, Spain

## Abstract

By using fluorescent labelling techniques, the distribution and dynamics of proteins can be measured within living cells, allowing to study in vivo the response of cells to a triggering event, such as DNA damage. In order to evaluate the reaction rate constants and to identify the proteins and reactions that are essential for the investigated process, mechanistic models are used, which often contain many proteins and associated parameters and are therefore underdetermined by the data. In order to establish criteria for assessing the significance of a model, we present here a systematic investigation of the information that can be reliably deduced from protein recruitment data, assuming that the complete set of reactions that affect the data of the considered protein species is not known. To this purpose, we study in detail models where one or two proteins that influence each other are recruited to a substrate. We show that in many cases the kind of interaction between the proteins can be deduced by analyzing the shape of the recruitment curves of one protein. Furthermore, we discuss in general in which cases it is possible to discriminate between different models and in which cases it is impossible based on the data. Finally, we argue that if different models fit experimental data equally well, conducting experiments with different protein concentrations would allow discrimination between the alternative models in many cases.

## Introduction

The technique of fluorescently labelling proteins made it possible to visualize cellular proteins and to measure their distribution and dynamics within the cell [1]. Via genetic engineering cells are manipulated such that they synthesise the protein of interest tagged with a fluorescent protein. The fluorescence signal of the cell is measured with a fluorescence microscope. Since the cells need not be fixed, it is possible to explore processes within living cells. The intensity of the fluorescence signal is proportional to the concentration of the tagged proteins, and its change in space and time reflects the changes of the protein concentrations. Comparing the signal within the region of interest, which has been treated in a special way (e.g., by irradiation), with an untreated region, allows to evaluate the relative concentration of proteins that are recruited to substrates (e.g., DNA) within the region of interest in response to the treatment. Examples for such experiments are the study of DNA repair processes after a damage [2], and cellular signalling subsequent to triggering light-inducible interactions [3]. If two or more different protein species are labelled, information about the interaction between these proteins can be obtained by measuring all their concentrations simultaneously.

Another application of fluorescently labelled proteins is FRAP (fluorescence recovery after photobleaching). FRAP allows the determination of the association and dissociation rate constants of proteins within cells by analyzing the curve of the recovering fluorescence signal after photobleaching. In contrast to the above-mentioned experiments, FRAP experiments are performed in equilibrium and not during the recruitment or response process. The theoretical background of FRAP has been extensively discussed [4], [5].

In the following, we focus on protein recruitment to a region in the cell following a triggering event, such as irradiation. There exist two kinds of mathematical approaches to deducing the binding and reaction rate constants from experimental data, the phenomenological and the mechanistic approach. In a phenomenological approach, mathematical functions (e.g., a monoexponential function) are fitted to protein recruitment data [6], [2], [7], [3], whereas mechanistic models use differential equations to describe the changes in the concentrations of activated or bound proteins in the region of interest [8], [9], [10], [11], [12], [13]. The aim of these mechanistic models often consists in evaluating rate constants, in identifying the proteins and reactions that are essential for the investigated process, and in obtaining evidence for processes that are not directly visible. Usually, protein diffusion is not included in such models, which is a good assumption as long as recruitment occurs at a considerably slower scale than diffusion, so that proteins do not become depleted near the site of recruitment.

Usually, mechanistic models assume that proteins are recruited in sequential order, and they contain many protein species and at least three times as many free parameters (association rate, dissociation rate, and initial concentration for each protein species), which means that the fit to the data may be good even when the underlying model is not valid. Such an approach is therefore only useful if the underlying model is well corroborated by other evidence or if competing models are so different that only one of them matches the data in spite of the large number of fit parameters. However, as Mueller et al. [14] pointed out often different models are able to describe experimental data equally well. For instance, Friedland et al. [15] noted that attachment and dissociation rates of Ku70/80 and DNA-PKcs involved in non-homologous end-joining cannot be derived unequivocally from the data. In the following, we will therefore assume that the complete set of reactions that affect the recruitment data of the considered protein species is not known. We will discuss in a systematic way which information can be deduced by comparing mechanistic protein recruitment models to data. To our knowledge, no such investigation exists yet in the literature. We will discuss a set of simple but realistic models consisting of three chemical species or less. We have chosen this number to keep the model as simple as possible but to still be able to describe situations in which one protein species influences another. We will show that in many cases it is possible to estimate the kind of interaction between two protein species by analyzing the shape of the recruitment curves. Furthermore, we will show in general in which cases the distinction between different models is possible and in which cases it is impossible based on the experimental data. Finally, we will show that if different models fit experimental data equally well in many cases conducting experiments with different protein concentrations would lead to a considerable improvement of the discriminability of the alternative models.

## Methods

The models we discuss consist of two or three chemical species. One of them is a substrate, such as a single strand break in the DNA, the other species are proteins binding to the substrate. We assume that these relations are composed of elementary reactions such as
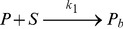
(1)and
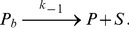
(2)In these formulas 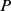 denotes a protein particle, 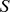 represents the substrate (e.g., the DNA), 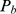 is the complex (e.g., a protein bound to DNA), and 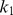 and 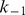 denote the forward and backward reaction rate constant. Assuming that the recruitment of the proteins happen on a slower time scale compared to diffusion and using the law of mass action this chemical reaction can be translated into the following set of differential equations for the concentrations of the chemical species:
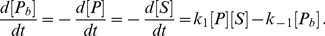
(3)Such a deterministic description is valid if the concentrations of the chemical species in the modeled volume are so high that the stochastic nature of the underlying processes is negligible. Even if this is not the case, the deterministic description is still useful as an approximation.

If the model includes more chemical species, the resulting set of coupled differential equations is in general not analytically solvable. However, for some important limiting cases analytical solutions exist. In particular, we consider the situation that the concentration of at least one chemical species is quasi constant. This situation arises for instance when there are few substrates (such as damaged DNA sites) but many proteins that could bind to them. In the following, the quasi constant approximation (QCA) will be applied to a protein species whenever the concentration of free proteins, 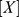, remains close to its initial value, 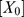, during the entire recruitment process and is always much larger than the concentration of bound proteins, 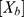. This means that 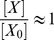 and 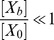. In the example above, the QCA can be applied if 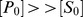. In the case 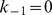, it leads to
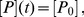
(4)

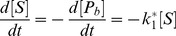
(5)with 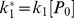.

Most of the models that we will discuss later consist of two protein species and the substrate. If the QCA is applied to both protein species, this will make all differential equations linear allowing to solve them analytically.

In order to compare curves resulting from different sets of parameters we normalize the curves to their steady state value. This is motivated by the fact that absolute values of the concentrations are usually not known in experiments, only relative changes in the concentrations can be evaluated. For the situation described by eq. (5), this leads to the functions
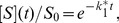
(6)

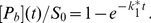
(7)In this case the system has only one free parameter, 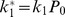.

In the following, we first discuss the situation of one protein species binding to the substrate, and then the case of two protein species binding to the substrate.

## 

### One protein one substrate model

The most simple model of protein recruitment includes a protein species, a substrate, and a chemical reaction for the binding of the protein to the substrate and its dissociation, as represented in eqs (1) to (3). This model can be solved analytically (see section A in Appendix S1). If the dissociation rate is negligible, the solution is
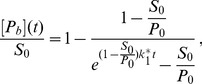
(8)whereas in general the solution is given by
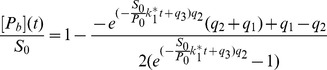
(9)with
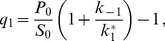
(10)

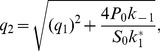
(11)

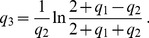
(12)The maximum 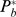, which corresponds to the limit 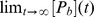, is given by
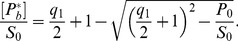
(13)


In the QCA, the differential equation and its solution simplify to
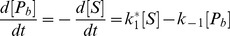
(14)with 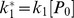 and
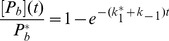
(15)with 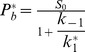.

Interestingly, this solution contains the two rate constants only in the combination 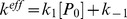. This means that if the concentration of the protein is much higher than that of the substrate, it is impossible to conclude from the normalized recruitment curve whether dissociation occurs. The fit gives only the combined value 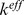.

Additionally, eq. (15) shows that an increase of the dissociation rate 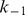 has an effect similar to an increase of the association rate 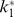. At first, this might seem counterintuitive. The reason for this effect is that increasing 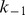 decreases the absolute maximum (obtained without normalization), while having only little effect on the slope of the not normalized curve at the beginning. The latter is due to the low concentration of 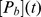 at the beginning. Both, the decreased maximum and the almost unchanged initial slope of the not normalized curves, lead to an increase of the slope of the normalized solution.

**Figure 1:**
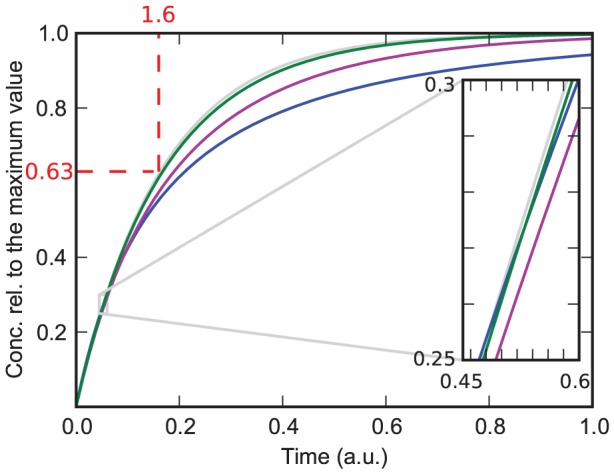
Effect of a variation of 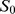 on the one protein one substrate model with dissociation. Coloured lines: Exact analytical solution (eq. (9)) normalized to 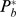 (eq. (13)) with 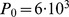, 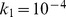, 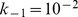. The value of 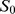 decreases from the lowest (blue) curve (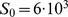) to the the highest (green) curve (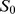 = 500). Grey line: QCA solution (eq. (15)) with 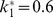 and 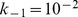. The inset shows the section in which the blue curve and the green curve cross, amplified by a factor of 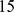. The dashed lines illustrate the parameter estimation described in the main text.

Figure 1 shows the normalized recruitment curves obtained for this model for different values of 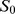. An important feature of this model is that curves resulting for different sets of the parameters can cross each other. This is impossible if dissociation is excluded. One can also see that with decreasing 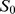 the curves of the analytical solution (eq. (9)) approach the QCA solution (eq. 15)).

In Figure 1 we also demonstrate how the parameter 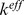 can be read off from the curve. First, we choose one point on the curve, for instance the initial point (as done in Figure 1). Then, we measure the difference of the concentration value at this point to the asymptotic value, which is 1 in our example. Then, we choose a second point that has a difference to the asymptotic concentration that is smaller by a factor 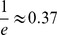. In our example, this point has the concentration value 0.63. The time interval between the two points then gives the inverse of the time constant. In our example, this time interval is 1.6, and we read off the result 
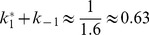
.

We conclude that if the QCA is applicable, the shape of the recruitment curve is monoexponential for the one protein one substrate model. The association and dissociation constant cannot be determined separately in this case, only the combination 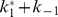 can be obtained by a fit to the normalized curves. When the QCA is not applicable, a monoexponential fit does not work, and a fit based on eq. (8) or (9) must be performed, giving the two parameters 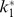 and 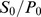 in the first case and the three parameters 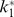, 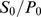, and 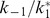 in the second case. Unfortunately, since the curves have a simple shape, a fit with eq. (8) will work very well even when the ratio 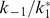 is in reality not small. Unless there is separate information on the value of 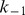 (obtained for instance from FRAP measurements) or on absolute concentration values, the value of 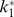 cannot be extracted from recruitment data. Nevertheless, the fit with eq. (8) gives an upper limit for 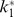, and assuming that the dissociation rate is not larger than 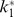, one can expect that the true value of 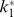 is somewhere between this upper limit and half this value. Thus at least the order of magnitude of 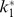 can be estimated. For instance, the parameter estimation of the grey curve shown in Figure 1 led to 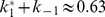. From this we can conclude that 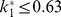. Additionally, if there is indication that at least half of the substrate places bind 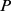 in equilibrium, it follows that 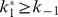. Then, the highest possible value for 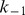 and the lowest possible value of 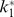 is 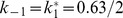.

Whenever a one protein one substrate model does not work, this indicates that additional molecules are involved in the recruitment process and influence the recruitment of the considered protein. This typically leads to recruitment curves that have a more complex shape than those shown in this subsection. In order to understand the different ways in which another molecule (which we take to be a second protein) can affect the recruitment dynamics of a given protein, we study in the next subsection the case of two proteins and a substrate.

### Two protein one substrate models

In this section we discuss models that consist of two proteins 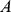 and 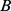 and a substrate 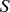. The influence of another protein can affect the association or the dissociation rate constant of a measured protein by either increasing or decreasing its rates. The analytical solution of the full model is complex and involves hypergeometric series. We therefore restrict ourselves to the QCA, which means that 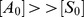 and 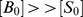. Since these conditions are satisfied in many biological situations, our results are widely applicable.

We denote with 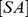, 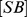, and 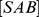 the concentrations of 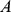 only, 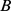 only, and the complex 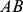 bound to the substrate 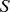. Again, 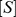 denotes the concentration of the unbound substrate. 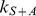 (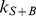) denotes the association rate constant of 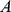 (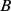) if 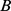 (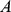) is not bound to the substrate, while 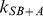 (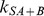) is the association rate constant if 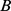 (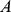) is already bound to the substrate. The corresponding dissociation rates are denoted with 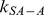, 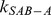, 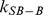 and 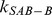.

The model consists of the following differential equations:
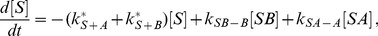
(16)

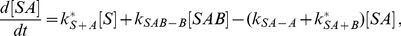
(17)

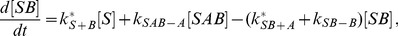
(18)

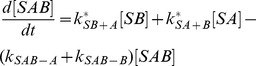
(19)with 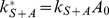, 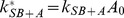, 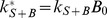 and 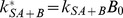. The sum of these four equations is zero, because the total concentration of bound and unbound substrate does not change.

In the following, we discuss specific model versions, which are nevertheless relevant in many situations and which give insights into the most general case by combining their insights. First, we discuss models in which all dissociation rate constants (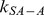, 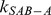, 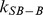, and 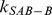) are assumed to be negligible and the association rate constants of at least one protein is influenced by the other protein (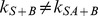). Then, we focus on models in which the dissociation rate constants are assumed to become important while we presume that the association rate constants are not influenced by the other protein (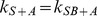 and 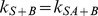).

### A. Protein 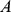 influences the association rate of protein 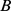


In many cases, association occurs on a much faster time scale than dissociation. When the recruitment curves are analyzed on a time scale where dissociation is not yet relevant, the dissociation rates 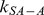, 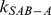, 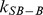, and 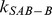 can be set to zero. Furthermore, we assume that the QCA is applicable and that the association rate of protein 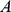 is independent of the binding status of protein 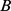 (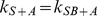), but that the association rate constant of 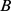 is influenced by the presence of 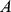 (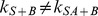). As we will show below, the more general case where each protein influences the association of the other can easily be deduced from the more special case studied in this subsection.

With the mentioned restrictions, the set of differential equations is given by
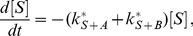
(20)

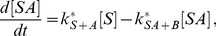
(21)

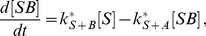
(22)

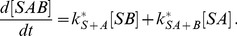
(23)The solution is (see section B in Appendix S1)
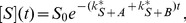
(24)

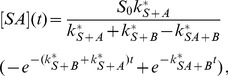
(25)

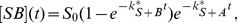
(26)

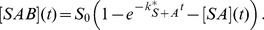
(27)Since the recruitment of 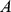 is described by the one protein one substrate model discussed in the previous section, we will focus in the following on protein 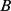. Unless 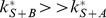, in which case 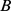 is recruited almost completely before 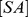 becomes non negligible, the recruitment curves of 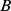 show an influence of protein 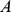. This influence is qualitatively different in the cases 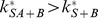 and 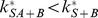, where 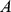 either increases or decreases the association rate of 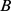. We therefore discuss these two cases separately.

#### A.1 Protein 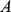 increases the association rate of 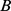


**Figure 2:**
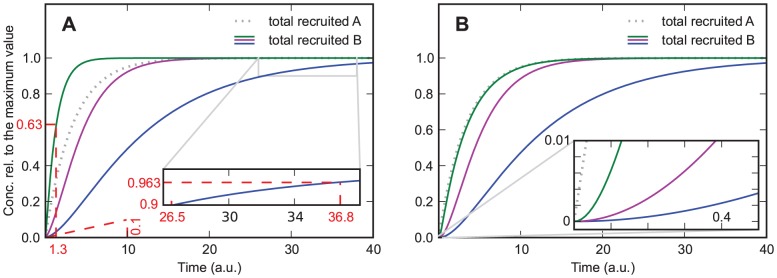
Model in which protein 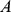 increases the association rate of protein 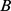. 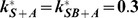 and 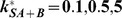 from bottom to top curve. A) 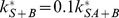. Solid lines are for protein 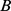, the dotted line is for protein 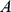, and the dashed lines are used for estimating the order of magnitude of the rate constants from the curves, giving 
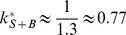
 for the steepest curve, and 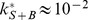 and 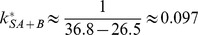 for the slowest curve. Depending on the parameters the curves show an increasing slope at the beginning. B) same parameters as in A) except 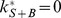. The curves always show an increasing slope at the beginning, which helps distinguishing this case from the one shown in Figure 2A. Additionally, for large values of 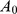 the recruitment curves of 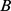 resemble the recruitment curve of 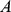, which is not the case if 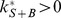.

If 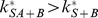, the association rate of 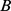 is increased by the presence of 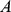. Figure 2 shows that this can lead to an initial increase of the slope of 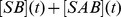 (i.e., the overall concentration of recruited protein 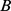). It can be shown analytically that the condition for such an initial increase is
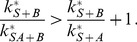
(28)Under experimental conditions, however, the initial increase of the slope will be concealed by noise unless the left hand side of eq. (28) exceeds the right hand side to a sufficient extent (depending on the noise level).

Even when the parameters are such that no upwards bend of the curve is visible (as in the green curve in Figure 2A), the slope of the curve is steeper than for a simple exponential function with exponent 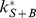. The special case 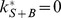 (i.e. 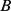 can not bind without 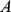) is characterized by a zero initial slope of the curve (Figure 2B). Admittedly it might be difficult to experimentally resolve this difference in the slope at 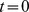. However, if several experiments with different values of 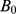 are performed, it is possible to identify the case 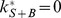 also by the signature that the curves approach with increasing 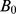 the asymptotic curve
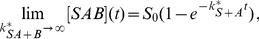
(29)which is the recruitment curve for protein 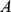. In contrast, in the case 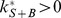 recruitment becomes ever faster with increasing 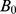 and is not limited by 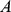 recruitment, see Figure 2A.

Approximate values of the parameters 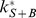 and 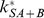 can be estimated by looking at the early and late stages of recruitment, which are dominated by 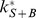 and 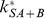, respectively. The initial slope of the curve is identical to 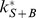, and it should be possible to extract this slope approximately from recruitment data. Alternatively, if 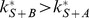, the time at which the curve reaches the value 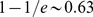 gives an impression of the order of magnitude of 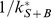. In the example shown in Figure 2A, the resulting estimate of 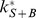 is 50 percent off the correct value. If protein 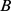 is recruited on a slower time scale than 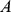, the value of 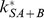 can be estimated by measuring the time it takes to decrease the distance to the asymptotic value 1 to 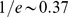 of its previous value (see inset of Figure 2A; a detailed explanation of parameter estimation can be found in section A).

An example of experimental data that show a slope that increases at the beginning is the recruitment curve of 53BP1 measured by Tobias et al. [2]. Indeed, it is known that the binding of 53BP1 depends on the binding of other proteins. In general, such an initial increase of the slope will be seen whenever the considered protein binds with a considerably increased rate when either another protein has become bound to the substrate or has performed a modification of the substrate, such as phosphorylation.

#### A.2 Protein 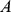 decreases the association rate of 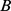


**Figure 3:**
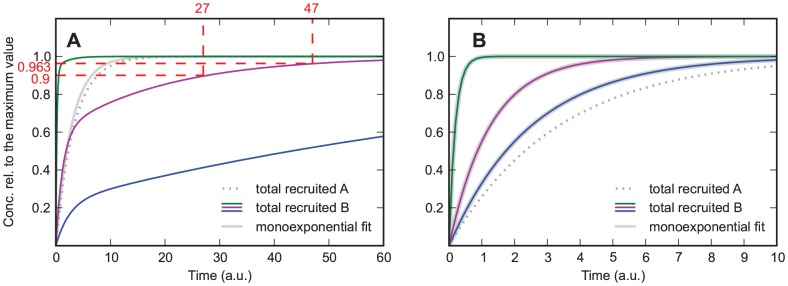
Model in which protein 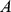 decreases the association rate of protein 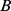. 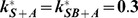 and 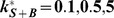 from bottom to top curve. A) 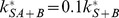. Solid lines are for protein 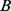, the dotted line is for protein 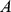. The grey line is a monoexponential fit. The dashed lines are used for estimating the rate constants from the curves, giving 
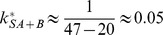
. After 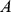 has risen to a high level, recruitment of 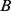 becomes much slower, and the slope of the recruitment curve of 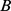 (purple curve) decreases faster than would be expected from a monoexponential curve that has a similar initial slope (grey curve). B) same parameters as in A) except 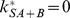. The curves always resemble a monoexponential function.

If 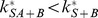, the association rate of 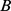 is decreased by the presence of 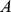. Figure 3A shows typical recruitment curves obtained in this case. After 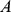 has risen to a high level, recruitment of 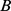 becomes much slower, and the slope of 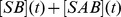 decreases faster than would be expected from a monoexponential curve that has a similar initial slope (grey curve). A three-parameter fit using the one protein one substrate model eq. (9) does not give good fits either (not shown). The reason for this is that the bend in 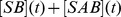 is caused by protein 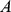 approaching saturation, and not by a depletion of binding sites for protein 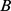, as in model eq. (9). The bend can therefore occur when 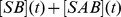 is still far from saturation.

An interesting special case is given by 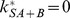 (Figure 3B). In this case protein 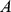 prevents the association of protein 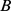, and the concentration of total recruited 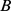 is given by:
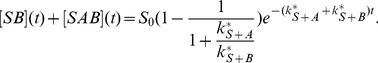
(30)According to eq. (30) the recruitment curve of 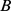 is monoexponential. Thus, if 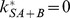, this model can not be distinguished from the model in which there is no influence on the protein. However, if the concentration of 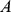 is measured as well, it is possible to distinguish between this model and the one in which there is no influence on the protein: If 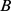 is blocked by 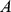, the absolute maximum of the recruitment curve of 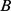 should decrease if 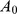 is increased (see Figure S1).

#### A.3 Protein 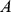 influences the association rate of 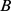 (dissociation is relevant)

If the dissociation rate of 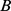 is relevant and all other conditions are the same as mentioned at the beginning of section B1 (QCA is applicable, 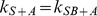, 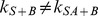, 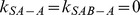, 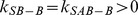), the set of differential equations is given by
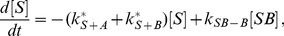
(31)

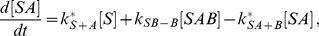
(32)

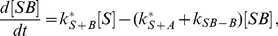
(33)

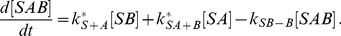
(34)with the solution (see section B in Appendix S1)
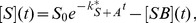
(35)

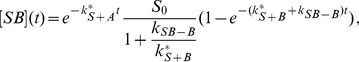
(36)

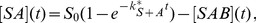
(37)

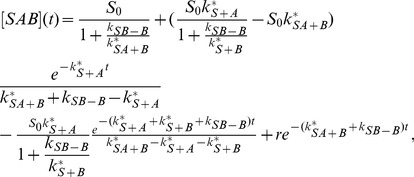
(38)

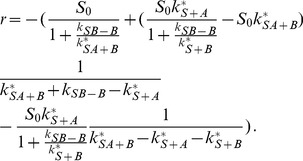
(39)


**Figure 4:**
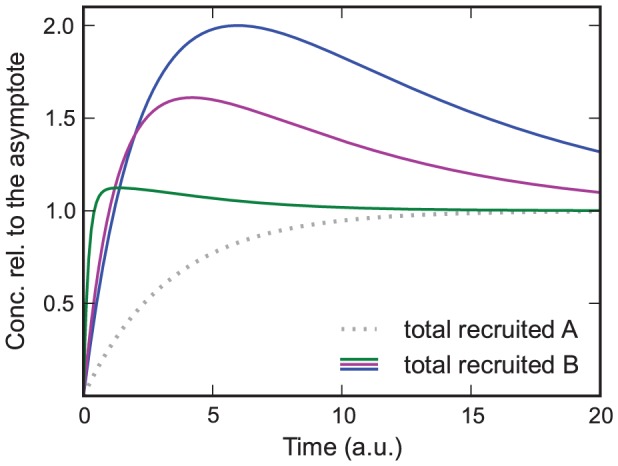
Model in which 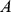 decreases the association rate of 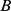 and in which dissociation is relevant. 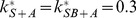 and 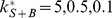 from green to blue curve; 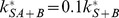 and 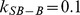. The curves are normalized to their asymptotic value at 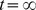. In contrast to the previous models, the recruitment curves of this model have a maximum that is larger than the plateau value if 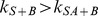 and 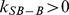.

The features of this model are the same as those in the previous models with the addition of a new feature, which can be seen in Figure 4. In contrast to the previous models, the recruitment curves of this model have a maximum that is larger than the plateau value if 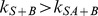 and 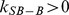. The reason for this feature is that at 
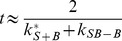
 the association and dissociation of 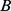 are equilibrated. But, since the concentration of recruited 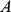 still increases, which in turn decreases the association rate of 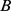, the equilibrium of 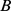 is shifted downwards. Interestingly the curve with the lowest 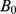 shows the highest peak, which can be explained by its lower asymptotic concentration at 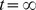, to which the curves were normalized.

If 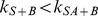 and 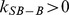, this can lead to an initial increase of the slope similar to the one observed in the previous model with 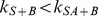 and 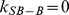. But, if 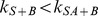 and 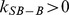, depending on how fast 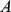 is recruited compared to 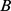 it is also possible that the curves show a slope that decreases too fast to be fitted by a monoexponential function, which is similar to the feature found in the previous model with 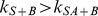 and 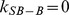. The reason for this is that at 
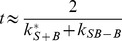
 the association and dissociation of 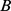 are equilibrated, but since the concentration of recruited 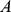 still increases and hence the association rate of 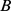 increases, the equilibrium is shifted upwards, which causes the slope that decreases too fast to be fitted by a monoexponential function. Since the two models with 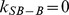 are able to reproduce only one of these two features (the initial increase of the slope or the slope that decreases too fast to be fitted by a monoexponential function), whereas the model with 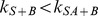 and 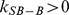 is able to reproduce both features, it is possible to discriminate between these three models, supposing that the underlying data are detailed enough.

#### A.4 Protein 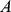 and 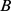 mutually influence their association rate

Next, we discuss the situation that both proteins influence the association of the other protein (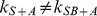, 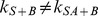). We still assume that dissociation is not relevant (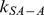, 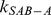, 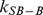, and 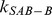 can be set to zero) and that the QCA is applicable. Equations (16) to (19) then become
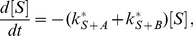
(40)

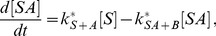
(41)

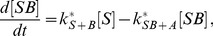
(42)

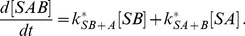
(43)


The solution is given by (see section B in Appendix S1):
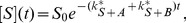
(44)

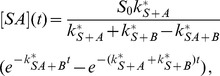
(45)

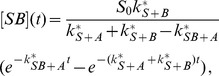
(46)

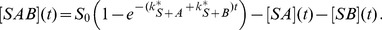
(47)The solution for 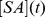 and 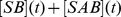 is identical to that obtained in the previous subsection, where 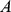 was not influenced by 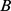. This means that the recruitment curve of 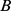 does not carry any information about whether 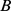 influences the association rate of 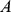. By exchanging 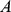 and 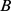, we can also conclude that the recruitment curve of 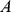 does not carry any information about whether 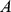 influences the association rate of 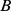. Only if both recruitment curves are measured is it possible to decide whether the influence is mutual.

An interesting special case is given by 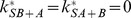. This means that the two proteins block each other. In this case, the normalized curves 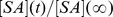 and 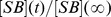 both are identical to 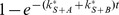, i.e., they saturate at the same time. If only normalized curves are considered, this situation, where the two proteins block each other, cannot be distinguished from the situation that the two proteins are recruited as a complex. In the latter case, the two recruitment curves are also identical.

However, if the proteins block each other, increasing the initial concentration of one of them will lead to an increase of the saturation value of this protein and to a decrease of the saturation value of the other protein. It should be possible to see this effect even when the absolute values of the concentrations cannot be obtained. If the two proteins are recruited as a complex, this effect does not occur. One can thus distinguish between the two situations by varying the protein concentrations.

### B. Protein 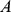 influences the dissociation rate of protein 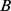


In general, a protein can not only affect the association rate of another protein, but also its dissociation rate. We therefore study in this subsection a model version where dissociation of one of the proteins (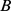) is assumed to become important and to be influenced by the other protein (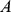). In order to see the effect of an influenced dissociation rate as clearly as possible, we assume that the association rate of neither protein is influenced by the other protein. This means that 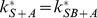 and 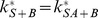. In contrast to the previous subsections, we now have 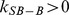 and 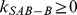, with 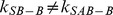. We still assume that dissociation of protein 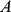 is not relevant (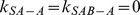) and that the QCA is applicable. Equations (16) to (19) then become
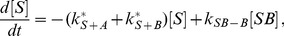
(48)

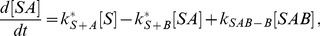
(49)

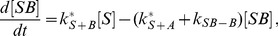
(50)

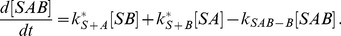
(51)


The solution is (see section B in Appendix S1)
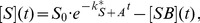
(52)

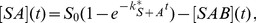
(53)

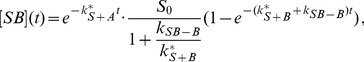
(54)

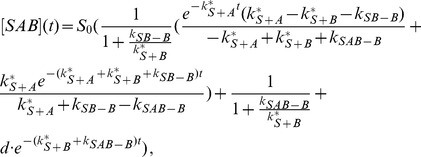
(55)with the constant
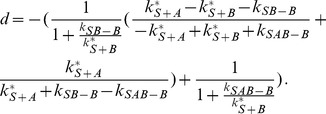
(56)


The expression eq. (54) for 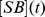 can be easily understood: the first factor describes the exponential decrease of the number of substrate sites at which 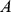 is not yet bound. The second factor is identical to eq. (15)) and describes 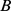 recruitment (with dissociation) to substrate sites where 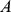 is not yet bound. For 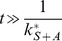, the concentration 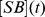 is very small, and most of the 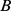 proteins bound to the substrate are bound in a complex with 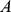. The saturation level of the complex is 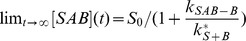 and is determined by the equilibrium between association and dissociation of 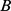. This result is valid because we have assumed that 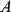 dissociation is not important, so that for large 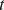 there is an 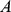 protein bound to virtually all substrate binding sites.

Depending on whether 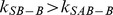 or the opposite is true, the recruitment curves show different features. These features are only visible if protein 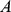 binds slowly compared to 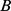 (i.e., 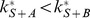). Otherwise, recruitment of 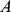 is already too advanced before 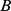 approaches its association-dissociation equilibrium, and the change in the dissociation rate of 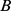 due to the presence of 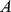 is hardly visible in the curves.

#### B.1 Protein 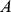 decreases the dissociation rate of 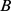


**Figure 5:**
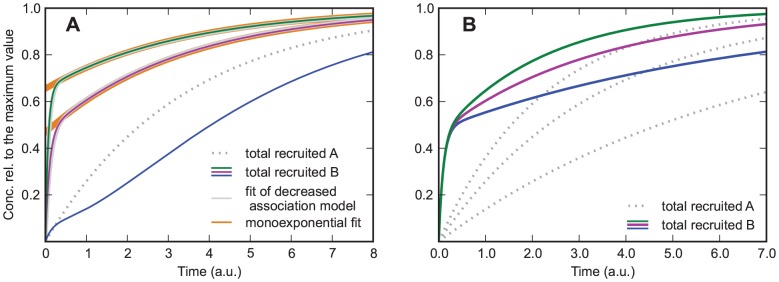
Model in which protein 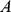 decreases the dissociation rate of protein 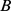. A) 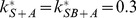, 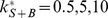 from bottom to top curve, 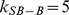, 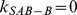. Grey curves: Fits of the model in which protein 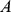 decreases the association rate of protein 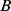. Orange curves: Monoexponential fit with an intercept. Decreasing 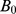 leads to a new feature, which is characterized by a decline of the slope followed by an increase. Since this feature can not occur in the model in which protein 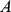 decreases the association rate, it allows distinguishing between the model in which protein 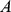 decreases the association rate and the model in which it decreases the dissociation rate. B) same parameters as in subfigure A, but 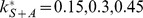 from bottom to top and 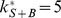. Despite of the variation in 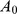, the time at which the bend occurs is the same for all curves.

If 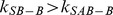, the recruitment curves counter-intuitively show a bend similar to the situation where 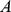 decreases the association rate of 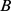, see Figure 5A. This bend occurs around 
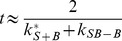
, when association and dissociation of 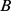 are nearly equilibrated at binding sites not occupied by 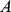. From this moment on, 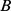 recruitment advances only slowly, with 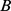 being almost at equilibrium with the recruited 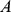 concentration. This equilibrium value increases as more 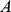 is recruited, because 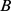 dissociation decreases. Since the shift in the equilibrium value is caused by 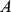, whose recruitment curve is monoexponential, the increase of the equilibrium value follows a monoexponential function as well. Thus, fitting a monoexponential function to the part of the curve after the bend leads to a good fit (Figure 5A orange curves). A similar bend was observed in the model where 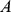 decreases the association rate of 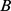. Thus, that model can be fitted well to the total recruited 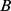 shown in Figure 5A (grey curves). Hence, another criterion is needed in order to distinguish between the model in which 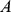 decreases the dissociation rate and the model in which it decreases the association rate.

If 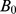 is lowered (Figure 5A bottom curve), the bend still occurs at the point at which 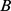 and 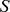 are equilibrated (
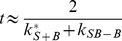
). But, because of the lower 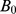, 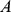 is recruited quicker compared to the time that is needed for 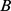 to be in equilibrium with the substrates to which 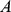 was bound before. Hence, the recruitment curve of the total recruited 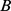 does not follow a monoexponential function after the bend. Instead, the slope of the curve increases after the bend because 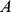 is recruited quicker compared to the recruitment of 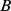 to substrates to which 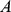 was bound before. Since from all models presented in this paper only in this one it is possible to see this feature (a decrease of the slope followed by an increase), it is useful to discriminate between alternative models.

Another feature which allows distinguishing between this model and the one in which 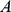 decreased the association rate is shown in Figure 5B. In these curves 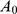 was varied by a factor of 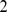 or less. The resulting recruitment curves of 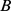 all show a bend at the same time and at the same concentration. If the concentration of 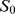 and 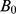 is varied as well, the bend still occurs at the same time but not at the same concentration. This would not be the case if the bend was a result of a decreased association rate. Since only a small variation of 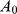 is needed for this criterion, the naturally occurring variation of 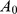 might be enough to apply this criterion even if the protein 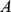 is not known.

#### B.2 Protein 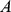 increases the dissociation rate of 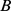


**Figure 6:**
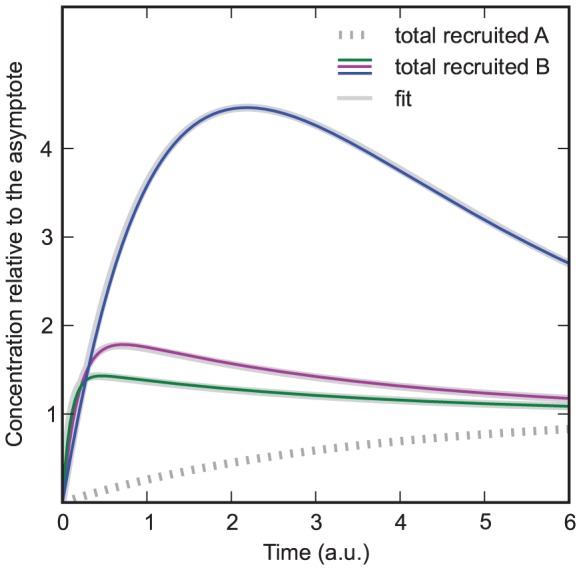
Model in which protein 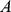 increases the dissociation rate of 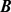. 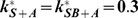, 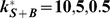 from green to blue curve, 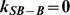 and 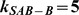. Grey curves: Fits of the model in which protein 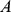 decreases the association rate of 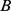 and in which dissociation is relevant (section B1.3).

If 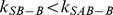 the recruitment curves of 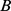 show an overshoot, which can be seen in Figure 6. The reason for this feature is that at 
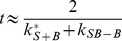
 the association and dissociation of 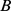 are equilibrated. But, since the concentration of recruited 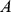 still increases, which in turn increases the dissociation rate of 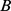, the equilibrium of 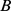 is shifted downwards. We discussed such an overshoot already in the model in which 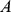 decreases the association rate and in which dissociation is relevant. Interestingly that model is able to fit the curves shown in Figure 6 quite well (grey curves). Nevertheless, it is possible to distinguish between both models in principle, because only the model in which 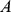 decreases the association rate and in which dissociation is relevant can reproduce two of the features presented in this paper, the overshoot and the slope that decreases too fast to be fitted by a monoexponential function, whereas the model with 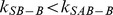 is able to reproduce the overshoot only.

## Discussion

**Figure 7:**
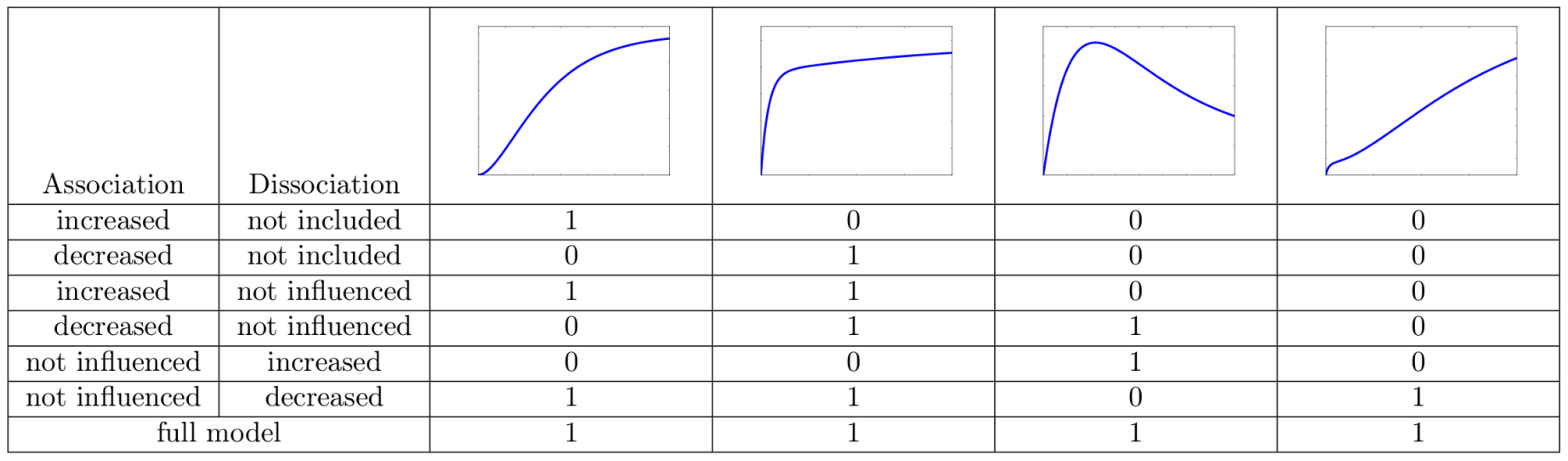
Comparison of the models.

In this study, we investigated simple mechanistic protein recruitment models and showed how the interaction between different proteins can be inferred by analyzing the shape of their recruitment curves. If one knows the recruitment curves of only one protein, in our models denoted by 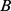, there are four features of the curves that indicate the influence of another protein on the proteins association or dissociation rate: The slope that increases at the beginning, the slope that decreases too fast to be fitted by a monoexponential function, the overshoot and the slope that decreases followed by an increase. Only the model in which protein 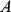 decreases the dissociation rate can lead to the latter feature. As summarized in Figure 7, the other three features can be caused by either a change of the association rate, a change of the dissociation rate, or a change of both rates. To resolve which is true, additional experiments have to be conducted in which the concentration of 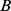 or, if possible, the concentration of 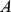 is varied.

In our models we assumed that either the association rate or the dissociation rate of one protein is influenced. This allowed us to solve the equations analytically. Since the association and dissociation of proteins often take place on different time scales, even in more complex cases (e.g. an influence on the association and the dissociation rate) the insights of this paper can be applied by dividing the recruitment curves into appropriate sections (i.e., a section in which dissociation is negligible and one where it is not).

Typical scenarios to which our models are applicable are those in which a protein is in need of another protein to fulfill its task, or in which one protein is a loading platform which is needed by others to bind to the substrate.

An interesting question is whether two proteins bind to a substrate as a preformed complex or each by its own (e.g. XRCC1 and its cofactor Ligase III, which are both involved in base excision repair). As we have shown, even if the recruitment curves of both proteins resemble each other, this does not necessarily mean that they bind as a preformed complex. The reason could be that the proteins bind in a sequential order and the protein which binds last has a much higher association rate than the other protein. Another explanation is that the proteins block each other, in which case the recruitment curves resemble each other as well. It is possible to distinguish between the sequential model and the other two models by analyzing the recruitment curve for small 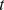. In order to distinguish between the model with the preformed complex and the one in which both proteins block each other within our framework, one would need to analyze the value of the maximum for different initial concentrations of the two proteins. Alternatively, one could use other methods, which are targeted at analyzing protein-protein interactions, to find out if the two proteins form a complex, for instance Foerster resonance energy transfer [16] or Co-immunoprecipitation [17].

In our models the dissociation of protein 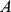 was not included. However, our findings are applicable to situations in which 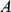 dissociates very quickly if the modification of the substrate (and thus the change of the rates of 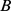) caused by 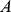 persists on a timescale that is long compared to the recruitment dynamics of protein 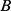.

To solve the differential equations we used the approximation that the protein concentrations are much higher than the substrate concentration. Even if this approximation is not valid, the recruitment curves will still show features that are qualitatively similar to those discussed in this study, and thus our findings are still useful. For instance, even if the quasi constant approximation is not valid, the model in which 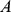 decreases the association rate of 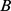 still is unable to reproduce the increasing slope and still is able to reproduce the slope that decreases too fast to be fitted by a monoexponential function (just as when the QCA is valid). In general, to find out which is the most simple model that is able to reproduce a given recruitment curve, it is necessary to try out all models that can not be excluded because of the shape of their recruitment curve.

For most of the methods presented in this paper it is not necessary to know which protein has an influence on the one that is measured. However, if this is known and the recruitment curve of the influencing protein is measured as well, this opens up much better ways to discriminate between alternative models, especially if the concentration of protein 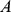 is known relative to the concentration of protein 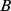. In this case, fitting the full model (eq. (16) to (19)) to the recruitment curves often reveals which parameters are negligible and which rates are not influenced.

## Supporting Information

Appendix S1 **Derivation of the analytical solutions.**
(PDF)

Figure S1
**Model in which protein** 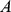 **decreases the association rate of protein** 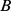 **: Special case** 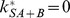 **.** 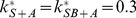 **(solid curves),** 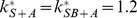 **(dashed curves),** 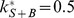 **and** 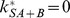 **.** The recruitment curve of 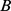 in the case with low 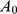 (solid blue curve) has a higher maximum as the recruitment curve of 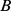 in the case with high 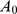 (dashed green curve). This feature helps distinguishing the model in which 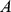 blocks 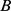 (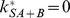) from the model in which there is no influence on 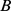. For further explanation see section B1.2 in the paper.(EPS)
