# Supplementary material for: Deducing Underlying Mechanisms from Protein Recruitment Data
Source: PLoS One. 2013 Jun 27;8(6):e66590. doi: 10.1371/journal.pone.0066590 (PMC3694963; doi:10.1371/journal.pone.0066590)
Supplement: Appendix S1 — Derivation of the analytical solutions. (PDF) [file pone.0066590.s001.pdf]

Appendix

A. One protein one substrate model

Derivation of equation (8)

If dissociation is negligible, the set of differential equations is given by:

$$\frac{d[P_b]}{dt} = -\frac{d[P]}{dt} = -\frac{d[S]}{dt} = k_1[P][S]\,. \tag{A1}$$

To solve this, we need the following equations, which describe that the total number of particles is preserved:

$$[P_b] = P_0 - [P] = S_0 - [S]\,. \tag{A2}$$

With this, we can write:

$$\frac{d[S]}{dt} = -k_1([S] + P_0 - S_0)[S]. \tag{A3}$$

Now, we use the method *separation of variables* to get:

$$\frac{d[S]}{[S]([S] + P_0 - S_0)} = -k_1 dt. \tag{A4}$$

From [1] we know the formula:

$$\int \frac{dx}{x(ax+b)} = -\frac{1}{b} ln \left| \frac{ax+b}{x} \right| + c \tag{A5}$$

with the constant of integration c.

Using eq. (A5) to integrate eq. (A4) leads to:

$$-\frac{1}{P_0 - S_0} ln \left| \frac{[S] + P_0 - S_0}{[S]} \right| = -k_1 t - c \tag{A6}$$

From eq. (A2) we know that $[S] + P_0 - S_0 = [P] \geq 0$, which allows rewriting eq. (A6) without the modulus function:

$$ln \frac{[S] + P_0 - S_0}{[S]} = (k_1 t + c)(P_0 - S_0) \tag{A7}$$

If we solve this equation for $[S]$ and choose the constant of integration in a way, which fullfills the condition $[S](t=0) = S_0$, we get the solution for $[S]$:

$$\frac{[S]}{S_0} = \frac{1 - \frac{S_0}{P_0}}{e^{(1-\frac{S_0}{P_0})k_1^* t} - \frac{S_0}{P_0}} \tag{A8}$$

Using eq. (A2) leads to the solution for $[P_b]$:

$$\frac{[P_b](t)}{S_0} = 1 - \frac{1 - \frac{S_0}{P_0}}{e^{(1-\frac{S_0}{P_0})k_1^* t} - \frac{S_0}{P_0}}\,, \tag{A9}$$

Derivation of equation (9)

If the dissociation rate is not negligible, the set of differential equations is given by:

$$\frac{d[P_b]}{dt} = -\frac{d[P]}{dt} = -\frac{d[S]}{dt} = k_1[P][S] - k_{-1}[P_b]\,. \tag{A10}$$

To solve this, we use the same steps as presented in the derivation of eq. (8). This leads to:

$$\frac{d[S]}{[S]^2 + (P_0 - S_0 + \frac{k_{-1}}{k_1})S - \frac{k_{-1}}{k_1}S_0} = -k_1 dt \tag{A11}$$

To integrate this equation we use the following formula from [1]:

$$\int \frac{dx}{ax^2 + bx + r} = \frac{1}{\sqrt{b^2 - 4ar}} ln \left| \frac{2ax + b - \sqrt{b^2 - 4ar}}{2ax + b + \sqrt{b^2 - 4ar}} \right| + c\,, \tag{A12}$$

with the constant of ingeration c.
After the integration of eq. (A11), we get:

$$\frac{1}{\sqrt{b^2 - 4r}} ln \left| \frac{2[S] + b - \sqrt{b^2 - 4r}}{2[S] + b + \sqrt{b^2 - 4r}} \right| = -k_1 t - c\,, \tag{A13}$$

with:

$$b = P_0 - S_0 + \frac{k_{-1}}{k_1}\,, \tag{A14}$$

$$r = -\frac{k_{-1}}{k_1}S_0\,. \tag{A15}$$

Next, we will show that $2[S] + b - \sqrt{b^2 - 4r} \geq 0$.
First, we need to know all possible values of $[S]$, for which we calculate the minimum S^*, which corresponds to the limit $\lim_{t\to\infty}[S](t)$. This limit has to fullfill the following equation:

$$\frac{dS^*}{dt} = k_1[P^*][S^*] - k_{-1}[P_b^*] = 0 \tag{A16}$$

From eq. (A2), we know that $P^* = S^* + P_0 - S_0$ and $P_b^* = S_0 - S^*$, which together with eq. (A16) leads to:

$$S^* = -\frac{P_0 - S_0 + \frac{k_1}{k_{-1}}}{2} + \sqrt{\frac{(P_0 - S_0 + \frac{k_1}{k_{-1}})^2}{4} + \frac{k_{-1}}{k_1}S_0} \tag{A17}$$

Since we always use the starting condition $P_b(t = 0) = 0$, the value of $\frac{d[S]}{dt}$ never will be positive and $[S]$ will monotonously decline from S_0 to S^*. This means that all possible values of $[S]$ are given by:

$$S_0 \geq [S] \geq S^* \tag{A18}$$

With this it can be shown that:

$$2[S] + b - \sqrt{b^2 - 4r} \geq 2S^* + b - \sqrt{b^2 - 4r} = 0\,. \tag{A19}$$

Now, we can write eq. (A13) without the modulus function:

$$\frac{1}{\sqrt{b^2 - 4r}} ln \frac{2[S] + b - \sqrt{b^2 - 4r}}{2[S] + b + \sqrt{b^2 - 4r}} = -k_1 t - c\,, \tag{A20}$$

Solving this equation for $[S]$ and using $[P_b] = S_0 - [S]$ leads to the solution presented in the paper (eq.(9)).

B. Two protein one substrate models

Derivation of equations (24)-(27) and (44)-(47)

We start with the following set of differential equations:

$$\frac{d[S]}{dt} = -(k_{S+A} + k_{S+B})[S]\,, \tag{A21}$$

$$\frac{d[SA]}{dt} = k_{S+A}[S] - k_{SA+B}[SA]\,, \tag{A22}$$

$$\frac{d[SB]}{dt} = k_{S+B}[S] - k_{S+A}[SB]\,, \tag{A23}$$

$$\frac{d[SAB]}{dt} = k_{S+A}[SB] + k_{SA+B}[SA]\,. \tag{A24}$$

The solution of $[S](t)$ is trivial:

$$[S](t) = S_0 e^{-(k_{S+A}+k_{S+B})t}\,. \tag{A25}$$

Now, we can rewrite equation (A23):

$$\frac{d[SB]}{dt} = -k_{S+A}[SB] + k_{S+B} S_0 e^{-(k_{S+A}+k_{S+B})t}\,, \tag{A26}$$

This is an inhomogeneous linear ordinary differential equation, which can be solved by the standard method *variation of parameters* to get the solution of $[SB](t)$ (equation (26)). The same steps apply to equation (A22) to get the solution of $[SA](t)$ (equation (25)). Now, it is possible to compute the solution of $[SAB](t)$ by integrating the right hand side of equation (A24).
The derivation of equations (44) to (47) consists of the same steps as presented above: First, $[S](t)$ is computed, which allows rewriting the differential equations of $[SA](t)$ and $[SB](t)$, which in turn can be solved by the method *variation of parameters.* Finally, it is possible to get the solution of $[SAB](t)$ by integration.

Derivation of equations (35)-(38) and (52)-(55)

We start with the following set of differential equations:

$$\frac{d[S]}{dt} = -(k_{S+A} + k_{S+B})[S] + k_{SB-B}[SB]\,, \tag{A27}$$

$$\frac{d[SA]}{dt} = k_{S+A}[S] + k_{SB-B}[SAB] - k_{SA+B}[SA]\,, \tag{A28}$$

$$\frac{d[SB]}{dt} = k_{S+B}[S] - (k_{S+A} + k_{SB-B})[SB]\,, \tag{A29}$$

$$\frac{d[SAB]}{dt} = k_{S+A}[SB] + k_{SA+B}[SA] - k_{SB-B}[SAB]\,. \tag{A30}$$

Adding the first to the third equation as well as adding the second to the fourth equation leads to:

$$\frac{d([S] + [SB])}{dt} = -k_{S+A}([S] + [SB])\,, \tag{A31}$$

$$\frac{d([SA] + [SAB])}{dt} = k_{S+A}([SA] + [SAB])\,. \tag{A32}$$

This set of differential equations is similar to the one presented in the methods section of the paper (equation (5)) if $[S]$ from equation (5) is substituted by $([S] + [SB])$ and $[P_b]$ from equation (5) is substituted by $([SA] + [SAB])$. Hence, the solution is given by equation (6) and (7) with the described substitutions:

$$[S](t) + [SB](t) = S_0 e^{-k_{S+A}t}\,, \tag{A33}$$

$$[SA](t) + [SAB](t) = S_0(1 - e^{-k_{S+A}t})\,. \tag{A34}$$

Solving equation (A33) for $[S](t)$ leads to equation (35), which allows rewriting equation (A29):

$$\frac{d[SB]}{dt} = k_{S+B}(S_0 e^{-k_{S+A}t} - [SB]) - (k_{S+A} + k_{SB-B})[SB]\,. \tag{A35}$$

Again, it is possible to use the method *variation of parameters* to solve this inhomogeneous differential equation, which leads to the solution for $[SB](t)$ (equation (36)).
Using the same steps, it is possible to compute the solution for $[SA](t)$ and $[SAB](t)$: First, equation (A34) has to be solved for $[SA](t)$, which leads to equation (37). Then, it is necessary to plug in the formula for $[SA](t)$ (equation (37)) and the solution of $[SB](t)$ (equation (36)) into equation (A30) to get a differential equation, in which only $[SAB](t)$ is unknown. This can be solved by using the *variation of parameters* method, which leads to the solution for $[SAB](t)$ (equation (38)).
The derivation of equations (52) to (55) follows the same steps as described above.

References

[1] Bronstein I, Semendjajew K (1989) Taschenbuch der Mathematik. Verlag Harri Deutsch.
